# High Smad7 marks inflammation in patients with chronic pouchitis

**DOI:** 10.3389/fimmu.2025.1549193

**Published:** 2025-03-03

**Authors:** Claudia Maresca, Andrea Iannucci, Marco Colella, Rachele Frascatani, Federica Laudisi, Elisabetta Lolli, Irene Marafini, Francesca Zorzi, Silvia Salvatori, Ivan Monteleone, Salvatore Bellinvia, Carmine Stolfi, Giovanni Monteleone

**Affiliations:** ^1^ Department of Systems Medicine, University of Rome “Tor Vergata”, Rome, Italy; ^2^ Department of Biomedicine and Prevention, University of Rome “Tor Vergata”, Rome, Italy; ^3^ Gastroenterology Unit, Azienda Ospedaliera Policlinico Tor Vergata, Rome, Italy; ^4^ PPM Services SA, Morbio Inferiore, Switzerland

**Keywords:** Crohn’s disease, ulcerative colitis, IBD, TGF, mucosal immunity

## Abstract

**Background and aim:**

Patients with ulcerative colitis (UC) undergoing colectomy with ileal-anal pouch anastomosis can develop chronic pouchitis (CP). Since treatment options are very limited for patients with CP, identification of factors/mechanisms that amplify the CP-associated inflammatory response could help develop novel treatments. We here assessed the expression of Smad7, an inhibitor of TGF-β1 signaling and positive regulator of gut inflammation, in CP.

**Methods:**

Mucosal samples were taken from the inflamed pouch of patients with CP, whose activity was evaluated by the modified Pouchitis Disease Activity Index (mPDAI). Controls included mucosal biopsy samples taken from the uninflamed pouch of patients with a history of CP and ileal samples taken from normal/inflamed pre-pouch of patients with CP and normal controls. Smad7 expression was assessed by Western blotting and immunofluorescence, and the Smad7-expressing lamina propria mononuclear cells (LPMCs) were evaluated by flow cytometry. Mucosal samples taken from the inflamed pouch of CP patients were cultured with a Smad7 antisense (AS) or sense oligonucleotide and TNF-α and interleukin (IL)-8 were evaluated by real-time PCR and ELISA.

**Results:**

Enhanced Smad7 expression was seen in the inflamed pouch of patients with CP compared to the normal or inflamed ileum of the same patients and the uninflamed pouch of patients with no pouchitis and normal controls. In the inflamed mucosa of patients with CP, Smad7 was more abundant in LPMCs, mainly in T lymphocytes. Knockdown of Smad7 in *ex vivo* mucosal explants taken from CP patients was associated with a reduction in TNF-α and IL-8 expression.

**Conclusions:**

High Smad7 occurs in the inflamed mucosa of patients with CP, further supporting the pathogenic role of Smad7 in the gut.

## Introduction

In the last decades, the advent of biologics and small molecules has contributed to reducing the rates of colectomy in patients with ulcerative colitis (UC) (1), although this is still needed in nearly 10% of patients at 10 years (2). Pouchitis is the most frequent complication after colectomy with ileal-anal pouch anastomosis (IPAA) (3, 4). Overall, 25% to 50% of UC patients who undergo IPAA surgery experience at least one episode of pouchitis within 10 years, which has a favorable response to antibiotics (5). Nonetheless, nearly one-fifth of these patients develop a chronic phenotype, either antibiotic-dependent or antibiotic-resistant, that requires further therapy, including biologics or small molecules. Unfortunately, more than 50% of these patients have an inadequate response to this treatment, which can lead to pouch failure and require pouch excision (5–7).

Although the pathogenesis of chronic pouchitis (CP) remains poorly characterized, it has been hypothesized that CP arises in individuals with genetic susceptibility as a result of an inadequate response of the mucosal immune system to the local microbiota (8–10). Identification of the factors/mechanisms that amplify pouchitis-associated inflammatory response could help develop novel treatments.

Accumulating evidence indicates that the inflamed gut of patients with UC and patients with Crohn’s disease (CD) contains elevated levels of Smad7, a protein that blocks TGF-β1 signaling, thus contributing to amplifying pathogenic immune responses (11, 12). In fact, the knockdown of Smad7 with a specific antisense oligonucleotide (AS) restores TGF-β1 signaling with the downstream effect of inhibiting inflammatory pathways both *in vitro* and in mouse models of colitis (12–14). These findings were consistent with the results of the Phase 1 and Phase 2 clinical trials showing that the administration of a pharmaceutical compound containing the Smad7 AS (termed Mongersen) in patients with active CD induced clinical and endoscopic improvement (15–17). However, subsequently, a large multicenter, randomized, double-blind, placebo-controlled, phase 3 trial was prematurely discontinued due to an interim analysis showing no effect of Mongersen on CD activity (18). However, further investigation of the pharmaceutical properties of Mongersen batches used in the phase 3 study revealed that most of them were unable to knock down Smad7 in cultured cells, highlighting the need to maintain consistent manufacturing requirements for clinical AS, as well as the potential benefits of *in vitro* bioassays as part of quality control (19).

This study aimed to investigate the expression of Smad7 in CP.

## Materials and methods

### Patients and samples

The modified pouchitis disease activity index (mPDAI) was used for the diagnosis of pouchitis (i.e. mPDAI ≥ 5) (20). Mucosal samples were taken from the inflamed pouch of active CP patients. Controls included mucosal biopsy samples taken from the uninflamed pouch of 6 patients without clinical/endoscopic evidence of pouchitis, from the terminal ileum of normal or inflamed, pre-pouch (pre-pouch ileitis) of patients with active CP and normal controls who underwent colonoscopy for colorectal carcinoma screening programs. Each patient who took part in the study gave their informed written consent and the study protocol was approved by the local Ethics Committee (Tor Vergata University Hospital, Rome, R.S. 58.23).

### Western blotting

Total proteins were extracted from mucosal biopsy samples. Samples were lysed on ice in a buffer containing 10 mM HEPES (pH 7.9), 10 mM potassium chloride (KCl), 0.1 mM ethylenediaminetetraacetic acid (EDTA), 0.2 mM ethylene glycol-bis (β-aminoethyl ether)- N,N,N’,N’-tetraacetic acid (EGTA), and 0.5% Nonidet P40 supplemented with 1 mM dithiothreitol (DTT), 10 mg/ml aprotinin, 10 mg/ml leupeptin, 1 mM phenylmethylsulphonyl fluoride (PMSF), 1 mM Na3VO4, and 1 mM sodium fluoride (NaF). The lysates were separated on sodium dodecyl sulfate (SDS)-polyacrylamide gels and transferred to nitrocellulose membranes using a Trans-Blot Turbo apparatus (Bio-Rad Laboratories, Hercules, CA). The membranes were incubated with antibodies anti-human Smad7 (1:1000, #MAB2029, R&D Systems, Minneapolis, MN), or anti-human-Vinculin (1:10000, #ab129002, Abcam, Cambridge, UK), followed by a secondary antibody conjugated to horseradish peroxidase (1:20000, # P0448, Dako, Santa Clara, CA). Membrane imaging was performed using chemiluminescence with the ChemiDoc Imaging System (Bio-Rad Laboratories).

### Immunofluorescence

Immunofluorescence was performed on frozen sections of ileal and pouch samples taken from CP patients, and ileal samples from CTR. The samples were embedded in a cryostat mounting medium (Neg–50, #6502, Epredia, Kalamazoo, Michigan), snap frozen, and stored at -80°C. Sections of mucosal biopsy samples were fixed with 4% paraformaldehyde for 10 min and permeabilized with 0.1% TritonX-100 for 20 min at room temperature. The sections were then blocked for 1 hour at room temperature (PBS, BSA 1%, goat serum 10%), and incubated overnight at 4°C with a mouse primary antibody against human Smad7 (1:150, R&D Systems), a rabbit primary antibody against human Smad7 (1:100, #37036, SAB, Greenbelt, Maryland) and with a mouse primary antibody against human EpCAM (1:1000, #2929, Cell signaling Technology, EuroClone, Milan, Italy). After washing with PBS 1X, the secondary goat anti-mouse Alexa488 antibody (1:1000, #A11017, Thermo Fisher Scientific, Waltham, MA), goat anti-rabbit Alexa488 antibody (1:2000, #A11008, Thermo Fisher Scientific) and goat anti-mouse Alexa568 antibody (1:2000, #A11004, Thermo Fisher Scientific), were applied for 1 hour at room temperature. After washing with PBS 1X, the sections were mounted using the prolonged gold antifade reagent with DAPI (#P36931, Thermo Fisher Scientific), and analyzed using the LEICA DMI4000 B microscope with the LEICA application suite software (V4.6.2) (Leica, Wetzlar, Germany).

### Cell isolation and culture

Lamina propria mononuclear cells (LPMCs) were isolated from mucosal biopsy samples using dithiothreitol (DTT)–ethylenediaminetetraacetic acid (EDTA) and through enzymatic digestion. Briefly, pieces of intestinal mucosa were washed in Hank’s balanced salt solution containing 1 mM DTT and antibiotics for 15 min at room temperature to remove mucus. The samples were then minced and incubated in Hank’s balanced salt solution containing 1 mM EDTA and antibiotics for 20 min at 37 °C to remove epithelial cells. After two washes in Hank’s balanced salt solution, the samples were incubated with Liberase TM (200 μg/Ml, #05401127001) and DNase I (200 μg/mL, #11284932001) (both from Roche Diagnostics GmbH, Mannheim, Germany), for 30 min at 37°C. After enzymatic digestion, mononuclear cells were collected.

To determine Smad7-expressing cells in CP LPMCs, the collected cells were analyzed by flow cytometry.

### Flow cytometry

CP LPMCs were stained with LIVE/DEAD cell viability assay (1:1000 for 10^6^ cells, #L34966A, Thermo Fisher Scientific). Cells were then washed, and stained for 30 min at room temperature with anti-human CD3-PerCP-Cyanine 5.5 (#45-0037-42, Thermo Fisher Scientific) CD45-APC-H7 (#641417, BD Biosciences, San Diego, CA), CD8-PE-Cy7 (#557746, BD Biosciences), CD56-AlexaFluor 647 (#318314, Biolegend, San Diego, CA), CD19-FITC (#345776, BD Biosciences), CD14-PE-Cy7 (#557742, BD Biosciences), CD68-APC (#333810, Biolegend), CD11c-PerCP-Cy 5.5 (#337210, Biolegend) (all used at 1:50 final dilution). After washing, cells were fixed and permeabilized with IC-Fixation Buffer (Bioscience) and Permeabilization Buffer (Thermo Fisher Scientific), respectively. Anti-human Smad7-PE (#orb485741, Biorbyt, Durham, North Carolina) was finally used to stain intracellular Smad7 for 30 min at room temperature. Appropriate isotype-matched controls were included. Gallios flow cytometer (Beckman Coulter, Brea, CA) was used for acquisition, and Kaluza software (Beckman Coulter) was used for analysis.

### *Ex vivo* organ cultures

Mucosal samples taken from CP patients were placed on steel grids in an organ culture chamber at 37°C in a 5% CO2/95% O2 atmosphere and cultured in RPMI 1640 medium. To determine whether Smad7 controls CP inflammatory response, the mucosal samples were either left untreated or transfected with a specific Smad7 AS or sense (control) oligonucleotide (both used a 10 µg/ml, GeneLink, Orlando, Florida) for 24 h using Opti-MEM medium and Lipofectamine 3000 reagent according to the manufacturer’s instructions (both from Life Technologies, Milan, Italy). The efficiency of transfection was determined by real-time PCR.

### Real-time PCR

Total RNA was extracted from CP LPMCs transfected with Smad7 sense or AS using the PureLink mRNA mini-kit (#12183025, Thermo Fisher Scientific). A constant amount of RNA (1 μg/sample) was retrotranscribed into complementary DNA (cDNA) using Oligo(dT) primers and M-MLV-reverse transcriptase (#28025021, Thermo Fisher Scientific). The cDNA was amplified using the following conditions: denaturation for 1 min at 95°C; annealing for 30 seconds at 59°C for human Smad7 and at 60°C for human β-actin; 30 seconds of extension at 72°C. RNA expression was calculated relative to the β-actin gene using the ΔΔCt algorithm. The primer sequences were as follows: Smad7 Fwd 5′-GCCCGACTTCTTCATGGTGT-3′, Rev 5′-TGCCGCTCCTTCAGTTTCTT-3′; β-actin Fwd 5’-AAGATGACCCAGATCATGTTTGAGACC-3’, Rev 5’-AGCCAGTCCAGACGCAGGAT-3’; TNF-α Fwd 5′-AGGCGGTGCTTGTTCCTCAG-3′, Rev 5′-GGCTACAGGCTTGTCACTCC-3′; IL-8 Fwd 5′-AGGAACCATCTCACTGTGTG-3′, Rev 5′-CCACTCTCAATCACTCTCAG -3′.

### Enzyme-linked immunosorbent assay

Cell-free supernatants of *ex vivo* organ cultures of mucosal biopsy samples taken from CP patients and transfected with sense or Smad7 AS were used to quantify extracellular TNF-α and IL-8 by enzyme-linked immunosorbent assay (ELISA) kits (#DTA00C and #D8000C respectively, both from R&D Systems). Absorbance readings were taken at 450 nm using a multimode detector DTX 880 (Beckman Coulter).

### Statistical analysis

Differences between groups were compared using the Student’s t-test or one-way ANOVA. The Pearson correlation coefficient was used to measure the linear correlation between the levels of Smad7 and mPDAI. All analyses were performed using Graph-Pad 9 software.

## Results

### Study population

Nineteen CP patients (6 female, 31.58%) who underwent endoscopic evaluation of the pouch were included in this study. The demographic and clinical characteristics of the patients are shown in **Table 1**. The median age of the patients was 47 years (range, 21-69 years). Two patients were smokers at the time of endoscopy, 5 were former smokers, and 12 were not smokers. Thirteen patients (68.42%) had an active clinical and endoscopic disease (mPDAI ≥ 5). At the time of mucosal sample collection, 8 patients were receiving no drug, 6 patients were taking biologics, and 5 patients were receiving antibiotic therapy.

**Table 1:** Demographic and clinical characteristics of patients.

Characteristics	Chronic pouchitis patients (N=19)
Female gender, n (%)	6 (31.58%)
Age (years), median [range]	47 [21-69]
Smokers	2 (10.53%)
Patients with clinical and endoscopic active disease, n (%)	13 (68.42%)
No therapy, n (%)	8 (42.11%)
Therapy with antibiotics, n (%)	5 (26.32%)
Therapy with biologic agents, n (%)	6 (31.58%)

### High expression of Smad7 in the inflamed pouch of CP patients

**Figure 1:**
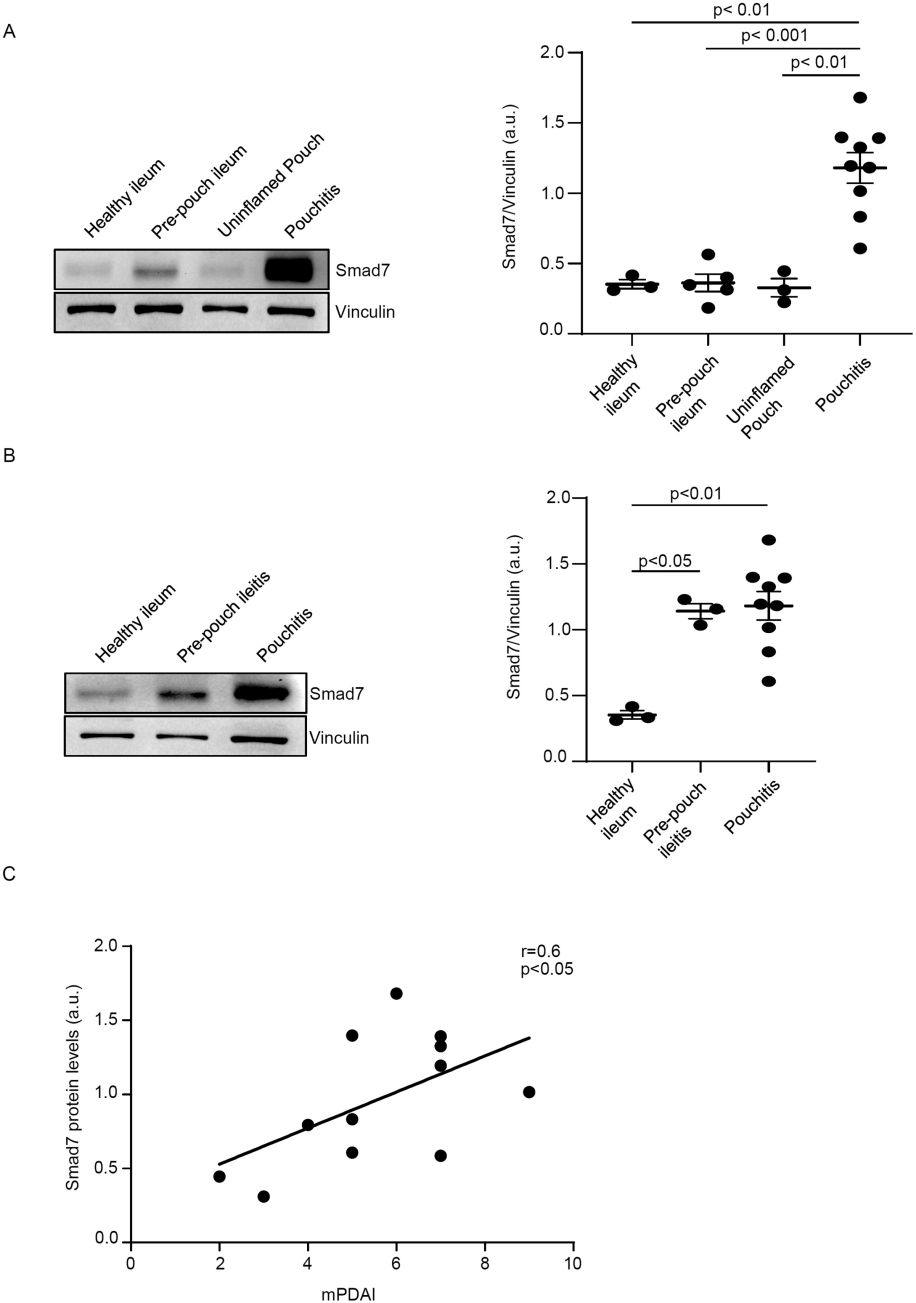
Smad7 protein expression is increased in chronic pouchitis (CP). **(A)** Representative Western blots showing Smad7 and Vinculin in total proteins extracted from mucosal samples of the inflamed pouch (pouchitis, n=9), pre-pouch ileum (n=5) of the same patient with CP, uninflamed pouch (n=3), and normal controls (healthy ileum, n=3). The right panel shows the quantitative analysis of Smad7/Vinculin ratio as measured by densitometry scanning of Western blots. Values are expressed in arbitrary units (a.u.) and indicate mean ± SEM of all samples. **(B)** Representative Western blots showing Smad7 and Vinculin in total proteins extracted from mucosal samples of the inflamed pouch (n=9), inflamed ileum (pre-pouch ileitis, n=3) of the same patients with CP, uninflamed pouch (n=3), and normal controls (n=3). The right panel shows the quantitative analysis of Smad7/Vinculin ratio as measured by densitometry scanning of Western blots. Values are expressed in arbitrary units (a.u.) and indicate mean ± SEM of all samples. **(C)**. Positive correlation between Smad7 protein expression in the pouch of 12 CP patients and Modified Pouchitis Disease Activity Index (mPDAI) evaluated by Pearson’s test coefficient (r = 0.6, p<0.05).

To determine whether the CP-associated inflammatory response is characterized by elevated expression of Smad7, we initially compared the protein levels of Smad7 in biopsy samples taken from 9 CP patients with those expressed in the uninflamed pouch of 3 patients with a history of CP, normal pre-pouch ileum (n=5) of patients with active CP and normal CTR ileal samples. Enhanced expression of Smad7 was observed in the inflamed pouch of patients with CP compared to the uninflamed pouch, normal pre-pouch ileum, and CTR (**Figure 1A**, **Supplementary Figure 1**). In 3 CP patients, endoscopy documented active lesions in the pre-pouch mucosa (pre-pouch ileitis). Smad7 protein expression in such samples was significantly higher than that seen in the normal ileum of the CTR and did not differ from that documented in the inflamed pouch of the same patients (**Figure 1B**, **Supplementary Figure 1**).

Next, we correlated Smad7 protein levels with mPDAI in 12 CP patients (9 with active disease and 3 with inactive disease). The data shown in **Figure 1C** indicate a positive correlation between the content of Smad7 and mPDAI.

### In the inflamed pouch, Smad7 is expressed in both the epithelial and lamina propria compartments

**Figure 2:**
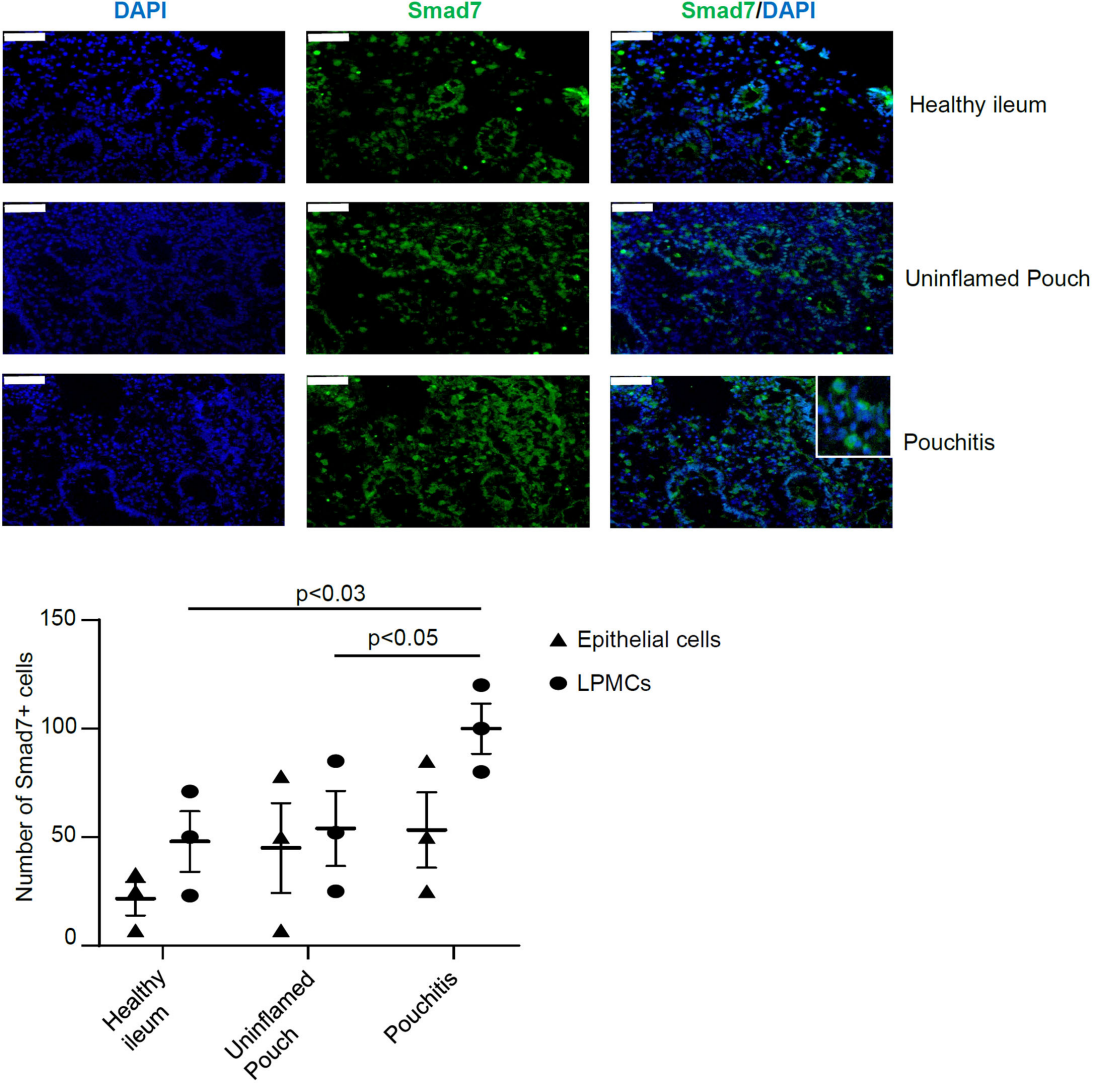
Smad7 is increased in the epithelial and lamina propria compartments of mucosal biopsies of patients with chronic pouchitis (CP). Representative images of immunofluorescence stainings of mucosal section of healthy ileum, uninflamed pouch, and inflamed pouch (pouchitis), which were analyzed for the expression of Smad7 (green), and DAPI (blue). The scale bars are 50 μm. The figure is representative of three separate experiments in which similar results were obtained. The lower scatter plot shows the number of Smad7-positive cells in epithelial cells and lamina propria mononuclear cells (LPMCs). Values indicate the mean ± SEM of 3 experiments.

Next, we determined which cells express Smad7 in the inflamed pouch of CP patients. For this purpose, we collected biopsies from clinically active CP patients with endoscopic evidence of lesions in the pouch and CTR and examined the expression of Smad7 by immunofluorescence. Smad7-positive cells were evident in both the epithelial and lamina propria compartments of biopsy samples taken from all groups (**Figure 2**). The expression of Smad7 in the epithelial compartment was confirmed through immunofluorescence co-staining with the epithelial cell marker EpCAM (**Supplementary Figure 2**). Quantification of the positive cells showed, however, that Smad7-expressing cells were more abundant in the lamina propria compartments of inflamed samples of CP patients than in CTR whereas no significant differences in terms of Smad7-positive epithelial cells were seen among the groups (**Figure 2**).

**Figure 3:**
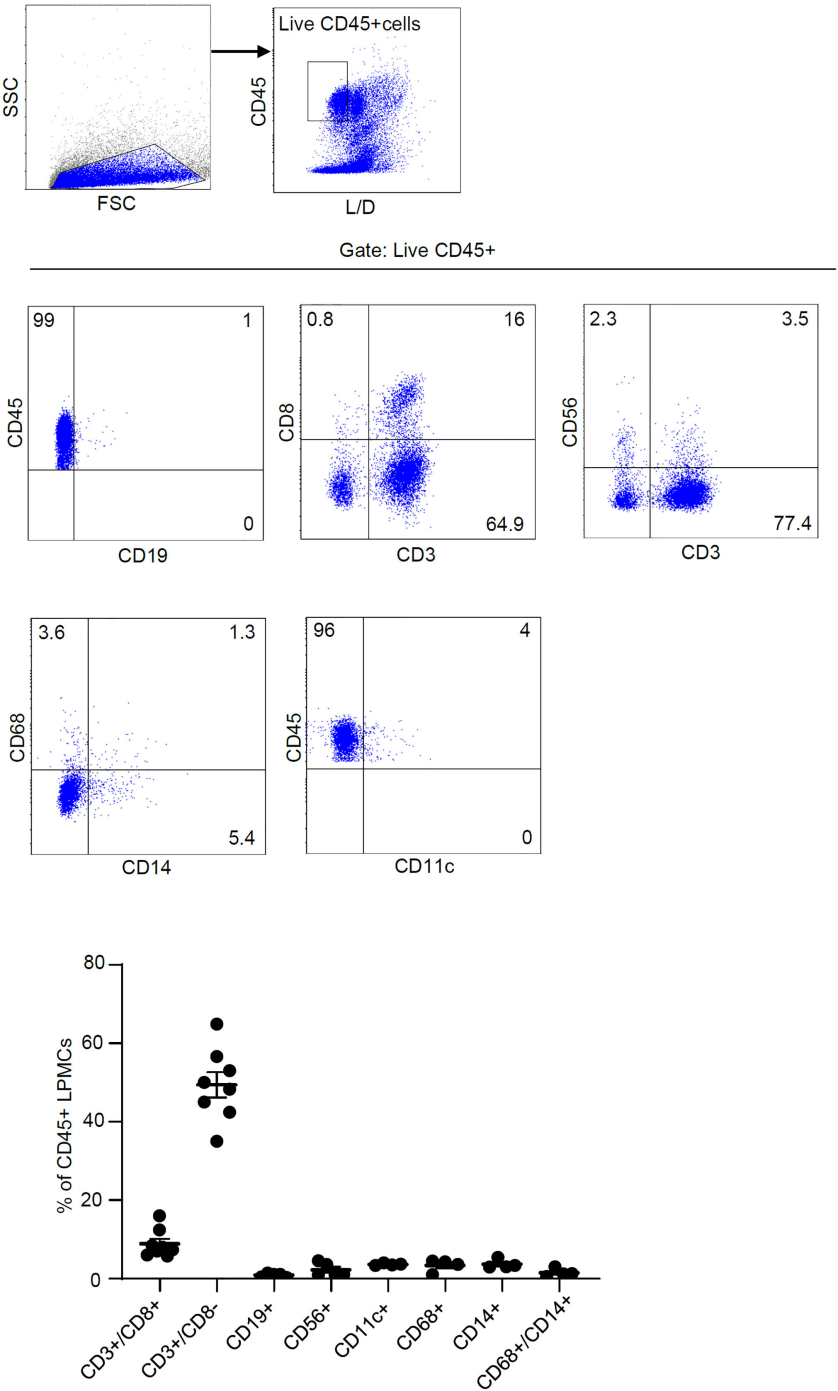
T lymphocytes are the main immune cell population in the mucosa of patients with chronic pouchitis (CP). Lamina propria mononuclear cells (LPMCs) were isolated from mucosal biopsy of 8 CP patients and the fractions of CD3+/CD8-, CD3+CD8+, CD19+, CD56+, CD11c+, CD68+, CD14+, and CD68+/CD14+ cells were evaluated in live, gated CD45-expressing cells. The data indicate mean ± SEM of 4-8 samples.

**Figure 4:**
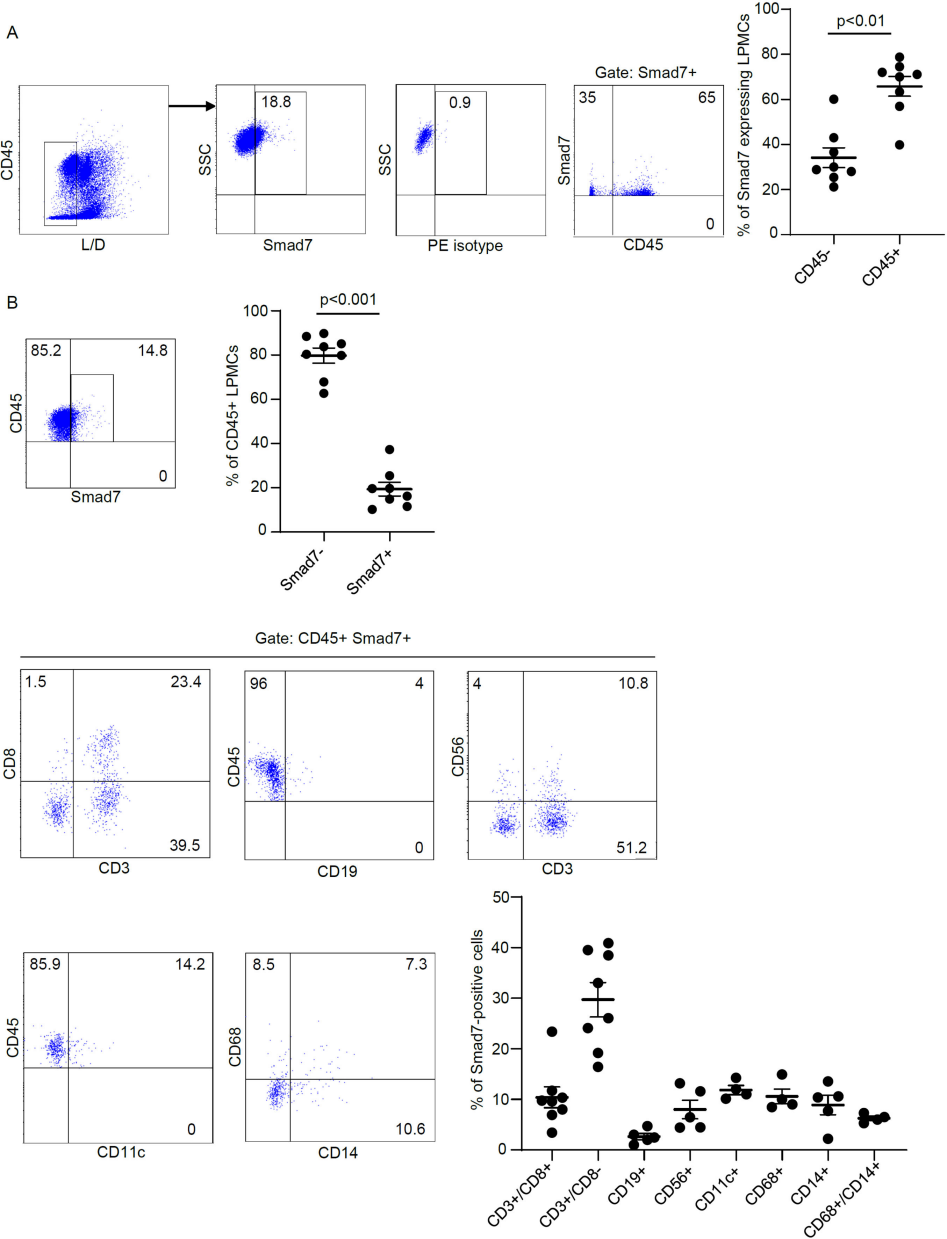
Characterization of Smad7-producing cells in LPMCs isolated from patients with chronic pouchitis (CP). **(A)** Representative dot-plots showing the fraction of Smad7-expressing live CD45+ and live CD45- cells in LPMC preparations isolated from mucosal biopsies of 8 CP patients and analyzed by flow cytometry. Staining with an isotype control antibody for Smad7 (PE isotype) is also shown. The right scatter plot indicates the mean ± SD of 4-8 experiments. Differences were analyzed using a two-tailed Student´s t-test. **(B)** Representative dot plots showing the percentage of live CD45+ Smad7+ cells expressing the indicated markers in LPMCs isolated from mucosal biopsies of CP patients. The scatter plots indicate the mean ± SEM of 4-8 experiments. Differences in the fraction of live CD45-positive cells expressing or not Smad7 were analyzed using a two-tailed Student´s t-test.

**Figure 5:**
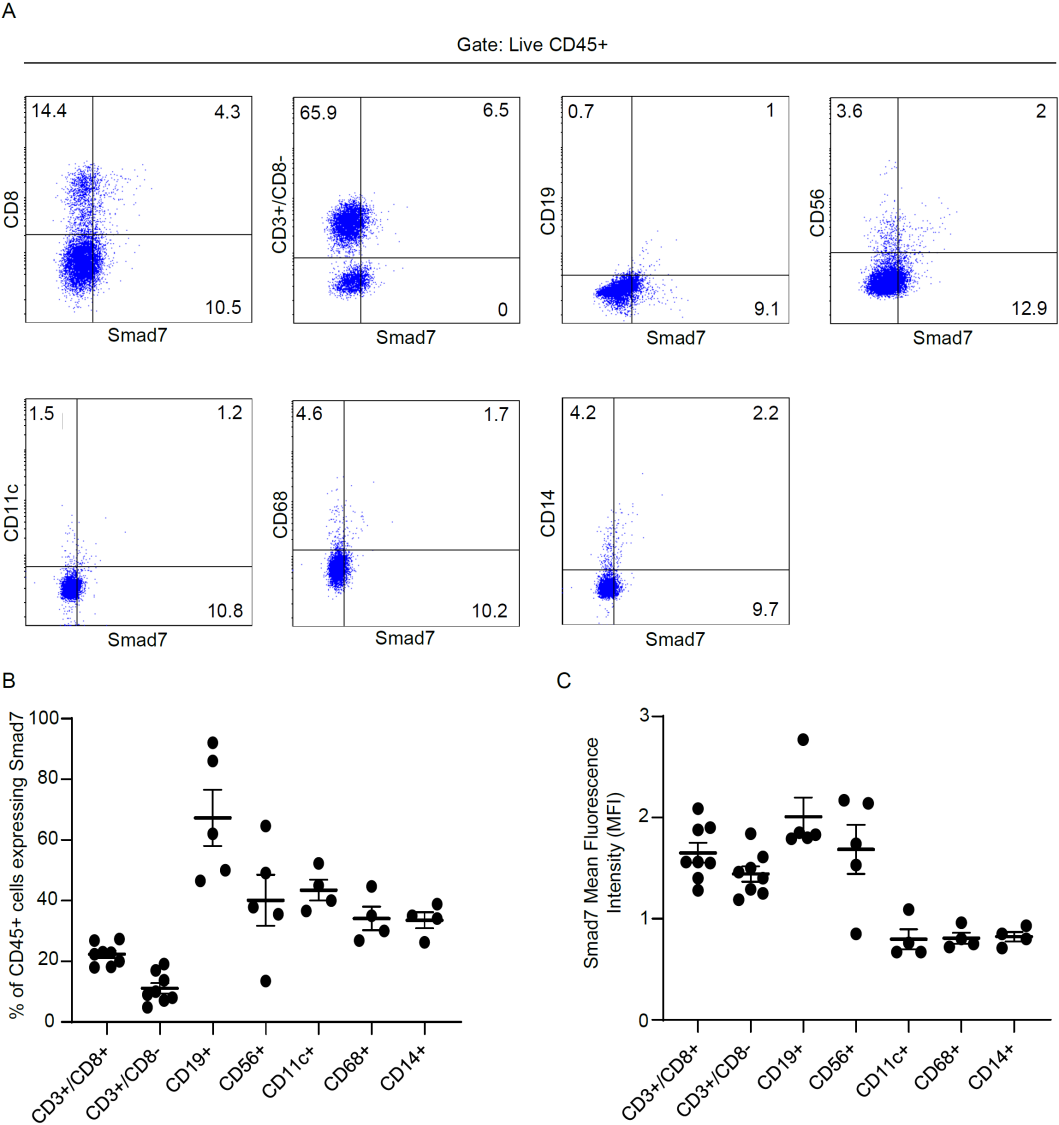
Analysis of Smad7 expression in immune cell subsets derived from LPMCs isolated from patients with chronic pouchitis (CP). **(A, B)** Lamina propria mononuclear cells (LPMCs) were isolated from mucosal biopsy of 8 CP patients and analyzed for the expression of Smad7 in live CD45-positive cells expressing CD3+/CD8+, CD3+/CD8-, CD19+, CD56+, CD11c+, CD68+ or CD14+. The data indicate mean ± SEM of 8 samples. Representative dot plots in which cells were gated on live CD45-positive cells and subsequently analyzed for the expression of Smad7, CD3+/CD8-, CD19+, CD56+, CD11c+, CD68+, CD14+ by flow cytometry. The bottom left scatter plot indicates the mean ± SEM of 4-8 experiments. The bottom right scatter **(C)** plot shows Smad7 Mean Fluorescence Intensity (MFI) in CD3+/CD8+, CD3+/CD8-, CD19+, CD56+, CD11c+, CD68+ or CD14+ cells.

To ascertain which cell types in the lamina propria compartment express Smad7, LPMCs isolated from inflamed CP samples were analyzed for Smad7 by flow cytometry. Initially, we assessed the fractions of CD3+/CD8-, CD3+CD8+, CD19+, CD56+, CD11c+, CD68+, and CD14+ cells in the inflamed pouch by gating on CD45-expressing cells. T lymphocytes, and mainly CD3+CD8- cells, were the dominant population in all the samples analyzed (**Figure 3**). Next, we gated Smad7-positive cells and assessed the percentages of CD45+ and CD45- cells. The majority of Smad7-expressing cells were CD45+ cells even though nearly one-third of Smad7-expressing cells were CD45 negative (**Figure 4A**). When the analysis was restricted to CD45+ cells, it was evident that one-fifth of them were positive for Smad7 (**Figure 4B**). Among the CD45+Smad7+ cells, CD3+CD8- T lymphocytes were the predominant population, even though the protein was variably expressed by the other cell types analyzed (**Figure 4B**). Further analysis of CD45+ LPMCs showed that more than 10% of CD3+CD8- T cells were positive for Smad7 (**Figures 5A, B**). Furthermore, Smad7 positivity was seen in more than 20% of CD3+CD8+ T cells, as well as in more than 30% of all the other cell populations analyzed (**Figures 5A, B**). Evaluation of the Smad7 mean fluorescence intensity (MFI) showed no significant differences among the various CD45+ LPMC subtypes (**Figure 5C**). In parallel, we assessed Smad7 expression in LPMCs isolated from biopsy samples taken from the terminal ileum of 2 healthy controls. However, Smad7-positive CD45-expressing LPMCs were barely detectable in the normal ileum (**Supplementary Figure 3**).

Collectively, these findings indicate that CP-associated inflammation is associated with elevated expression of Smad7 in immune and non-immune cells.

### Knockdown of Smad7 in CP *ex vivo* mucosal explants is associated with changes in the expression of inflammatory molecules

**Figure 6:**
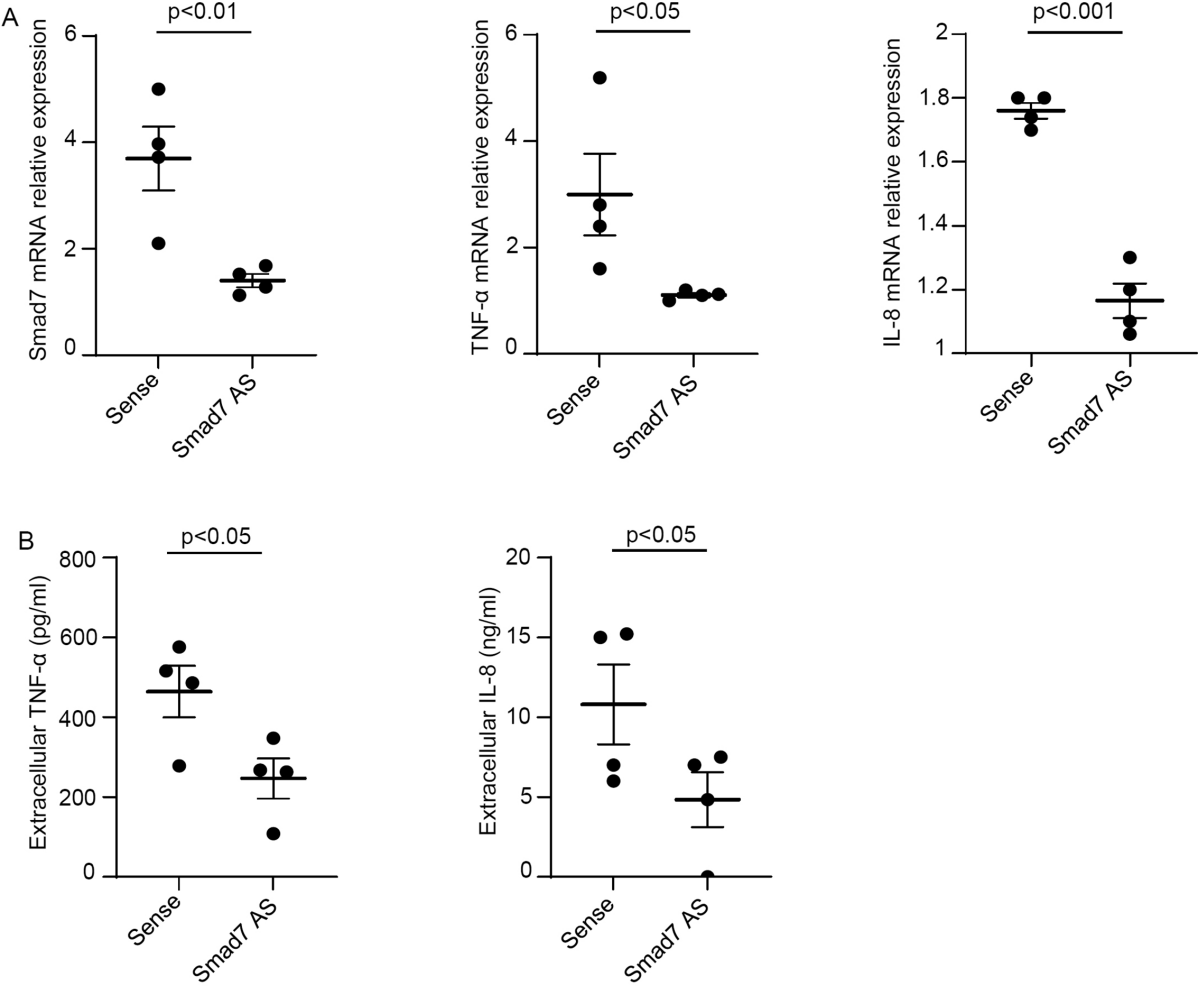
Smad7 knockdown in *ex vivo* organ cultures of mucosal biopsies of patients with chronic pouchitis (CP) hampers the production of inflammatory cytokines. **(A)** Mucosal biopsies were transfected with Smad7 sense oligonucleotide (Sense) or Smad7 antisense oligonucleotide (Smad7 AS) (both used at 10 µg/ml) for 24 h. Smad7, TNF-α and IL-8 RNA transcripts were evaluated by real-time polymerase chain reaction. Levels were normalized to β-actin. Values indicate the mean ± SEM of four independent experiments where samples taken from four different patients were used. Differences were analyzed using a two-tailed Student´s t-test. **(B)** Levels of TNF-α and IL-8 in cell-free supernatants of *ex vivo* organ cultures of mucosal biopsy samples taken from CP patients and transfected with sense or Smad7 AS for 24 h as assessed by ELISA. Values indicate the mean ± SEM of four independent experiments where samples taken from four different patients were used. Differences were analyzed using a two-tailed Student´s t-test.

To functionally link the high Smad7 with the ongoing mucosal inflammation in CP, we inhibited Smad7 in *ex vivo* mucosal explants of patients with CP with a well-characterized AS and evaluated the changes in the expression of TNF-α and IL-8, the latter being involved in the recruitment of neutrophils to the inflamed pouch (21, 22). Smad7 knockdown resulted in a significant down-regulation of TNF-α and IL-8 at both mRNA and protein levels (**Figures 6A, B**).

## Discussion

This study was undertaken to determine whether Smad7 is over-expressed in CP. We report up-regulation of Smad7 in the inflamed pouch of CP patients compared to the normal ileum of healthy individuals and patients with CP, and the uninflamed pouch of patients with a history of CP. The findings are in line with data from our initial study aimed at exploring the function of Smad7 in IBD, which included the analysis of Smad7 in two samples taken from CP patients: in both samples, Smad7 expression was greater than that found in the normal, unaffected colon (12). A small subgroup of our CP population had pre-pouch CD-like ileitis (23), as the endoscopic lesions extended up to the pre-pouch ileum. In such samples, Smad7 expression was similar to that documented in the inflamed pouch of the same patients. These findings, together with the demonstration that in patients with CP there was a good correlation between the Smad7 content in the pouch and the mPDAI, reinforce the notion that the ongoing mucosal inflammation rather than other variables (e.g. current therapy) is the driving force for Smad7 induction in the gut.

We also collected biopsy samples from the inflamed and uninflamed pouch and normal terminal ileum to localize Smad7-positive cells by immunofluorescence. In all the subgroups analyzed, Smad7-expressing cells were evident in both the epithelial and lamina propria compartments, but in each of these compartments, the number of Smad7-positive cells was higher in the inflamed pouch than in the controls. Next, we characterized the lamina propria cell sources of Smad7. Since the number of cells obtained from tiny endoscopic biopsies was not sufficient to make further comparisons among the various groups, we focused our analysis on cells isolated from the inflamed pouch. Flow cytometry analysis of CP LPMCs showed that Smad7 was mainly expressed by CD45+ cells, and among these, T lymphocytes were the main source, even though Smad7 was evident in virtually all types of immune cells analyzed, including myeloid cells, B cells, and NK cells. In contrast, Smad7 MFI in T lymphocytes did not differ from that measured in other cell types and the analysis of Smad7 in single cell types revealed that only one-third of T cells were positive. Altogether these data suggest that the upregulation of Smad7 in active CP reflects, at least in part, the increased infiltration of T lymphocytes in the inflamed mucosa of the pouch rather than a specific induction of the protein in such cell types. Smad7 was also detectable in nearly one-third of CD45-negative cells. The LPMC isolation procedure allows the recovery of non-immune cells, including various subsets of stromal cells, and endothelial cells, which are known to express Smad7 in other systems (24, 25). Thus, it is conceivable that these cell types contribute to the positivity of Smad7 in the CD45-negative lamina propria compartment of patients with active CP. Studies are now ongoing to address this issue.

Knockdown of Smad7 in CP mucosal explants with a specific AS led to a significant down-regulation of TNF-α and IL-8. This data could be pathogenically relevant as both cytokines are over-produced in CP and are supposed to amplify the mucosal inflammation (26). The molecular mechanism by which Smad7 controls the expression of such inflammatory molecules remains unclear even though it is conceivable that the documented changes in TNF-α and IL-8 production can reflect the modulatory effects of Smad7 on various signaling pathways controlling inflammatory gene expression. In this context, for instance, we previously documented a positive effect of Smad7 on the activation of NF-kB, a transcription factor that up-regulates the production of inflammatory cytokines and contributes to the propagation of mucosal inflammation (27, 28). Additionally, the Smad7-mediated abrogation of TGF-β1 activity could result in changes in the function of MAP kinases, another signaling pathway that ultimately influences cytokine/chemokine secretion (29).

Altogether the above findings confirm and expand on previous data supporting the inflammatory role of Smad7 in the gut (12–14), and raise the possibility that Smad7 is a target for the treatment of patients with chronic pouchitis. However, the failure of oral Mongersen in CD patients suggests the need for a better definition of potential candidates for such a treatment as well as the optimization of the pharmaceutical compounds containing the Smad7 AS before future clinical trials (18).

We are aware that the relatively small sample size can represent a limitation of this study, although there was a noticeable difference between CP patients and controls in terms of Smad7. The fact that Smad7 is expressed in epithelial cells other than LPMCs raises the possibility that Smad7 plays a role in the control of epithelial cell behavior. However, we experienced technical difficulties in setting experiments with epithelial cells isolated from CP and assessing the function of Smad7. Although the inflammatory role of Smad7 was validated by assessing the expression of two cytokines, classically associated with gut inflammation, in cultures of mucosal samples treated with Smad7 AS, further work is needed to fully evaluate the contribution of Smad7 in the propagation of CP-associated inflammation.

In conclusion, this study shows that Smad7 is over-expressed in the inflamed mucosa of patients with CP, further supporting the pathogenic role of Smad7 in the gut.

## Data Availability

The original contributions presented in the study are included in the article/**Supplementary Material**, further inquiries can be directed to the corresponding author/s.

## Supplementary material

Supplementary Figure 1 Uncropped blots for Smad7 and vinculin representing those depicted in **Figures 1A, B** of the main text as indicated.

Supplementary Figure 2 Representative images of double-immunofluorescence staining of mucosal biopsy samples taken from the inflamed pouch (pouchitis) showing Smad7 (green), EpCAM (red), and DAPI (blue). The scale bars are 10 μm. The figure is representative of three separate experiments in which similar results were obtained.

Supplementary Figure 3 Representative dot-plots showing the fractions of live Smad7-positive CD45-expressing cells in LPMC preparations isolated from biopsy samples taken from the terminal ileum of a healthy control. Staining with an isotype control antibody for Smad7 (PE isotype) is also shown. The example is representative of 2 experiments using cells of 2 normal controls.
