# Supplementary figures and images for: High Smad7 marks inflammation in patients with chronic pouchitis
Source: Front Immunol. 2025 Mar 3;16:1549193. doi: 10.3389/fimmu.2025.1549193 (PMC11911167; doi:10.3389/fimmu.2025.1549193)

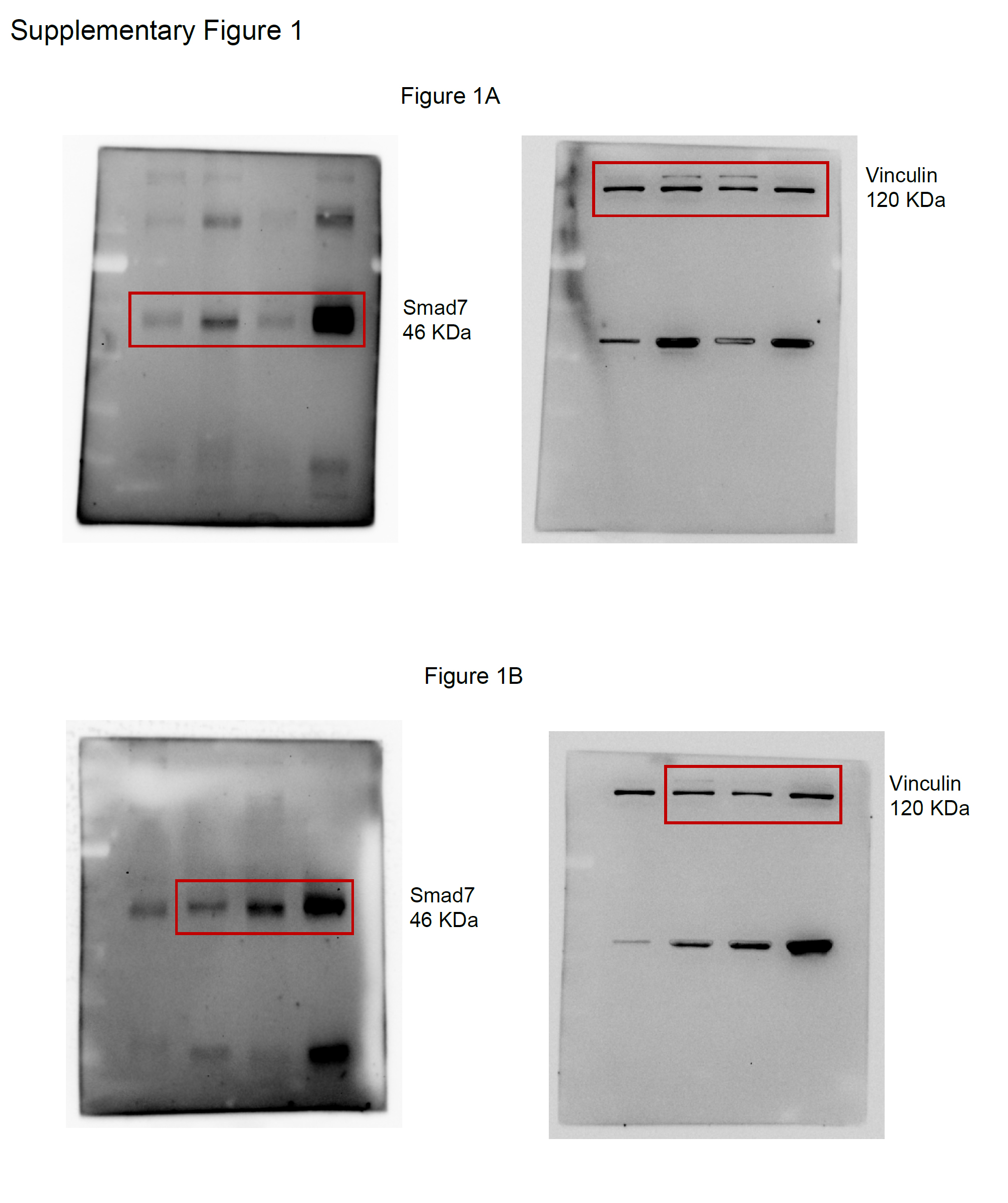

Supplement: Supplementary Figure 1 — Uncropped blots for Smad7 and vinculin representing those depicted in Figures 1A, B of the main text as indicated. [file Image1.jpeg]

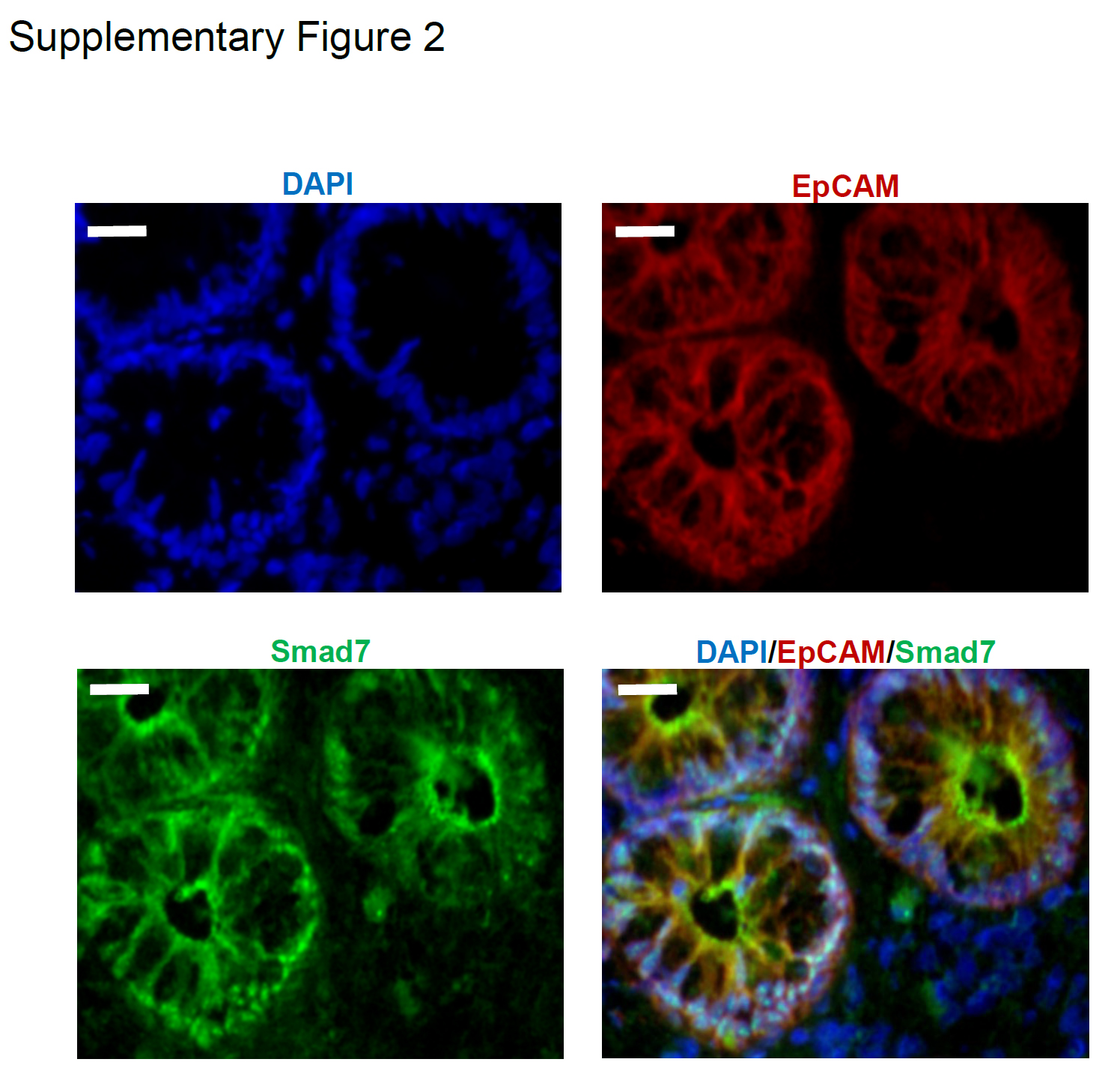

Supplement: Supplementary Figure 2 — Representative images of double-immunofluorescence staining of mucosal biopsy samples taken from the inflamed pouch (pouchitis) showing Smad7 (green), EpCAM (red), and DAPI (blue). The scale bars are 10 μm. The figure is representative of three separate experiments in which similar results were obtained. [file Image2.jpeg]

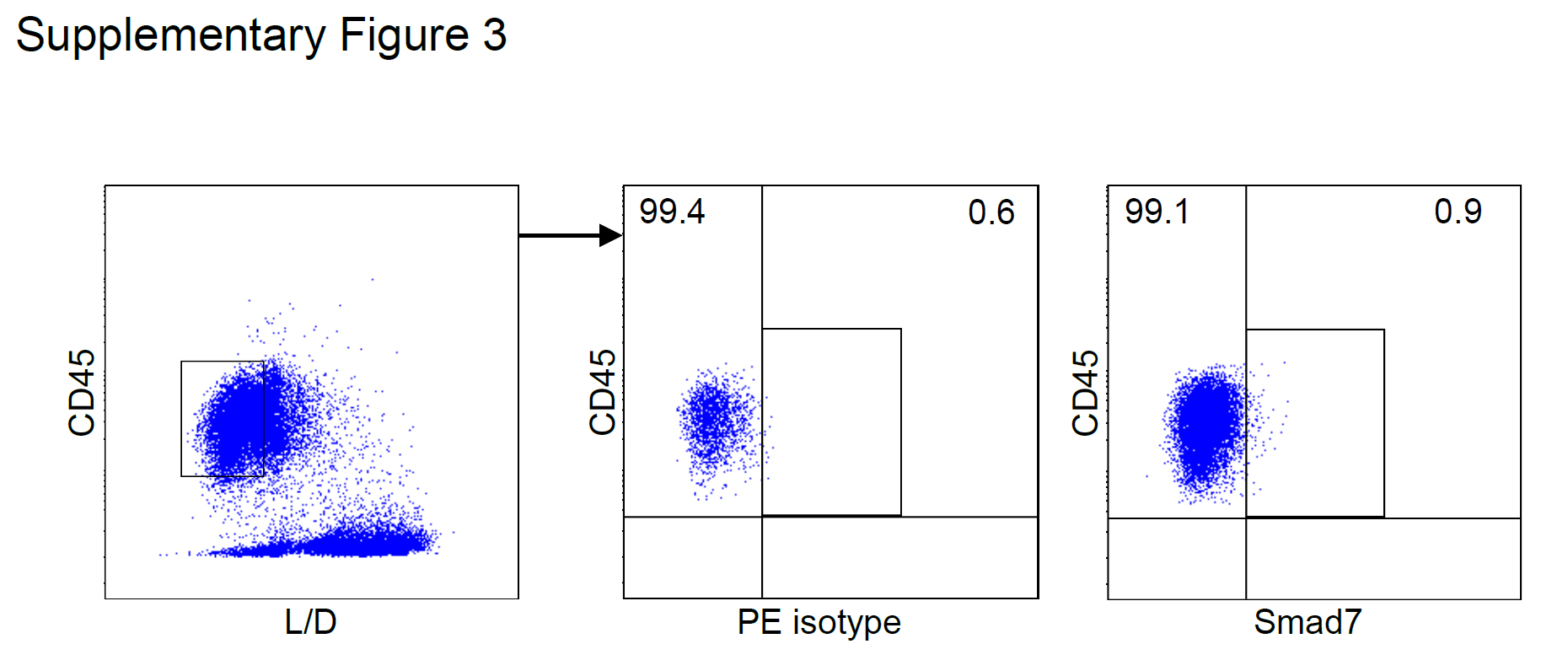

Supplement: Supplementary Figure 3 — Representative dot-plots showing the fractions of live Smad7-positive CD45-expressing cells in LPMC preparations isolated from biopsy samples taken from the terminal ileum of a healthy control. Staining with an isotype control antibody for Smad7 (PE isotype) is also shown. The example is representative of 2 experiments using cells of 2 normal controls. [file Image3.jpeg]
